# Optimization of print parameters for batch and continuous manufacturing of three-dimensional (3D) printed dosage forms using artificial intelligence and machine learning

**DOI:** 10.1007/s13346-025-02006-4

**Published:** 2025-11-04

**Authors:** Kshitij Chitnis, Yizhou Lu, Benjamin Rhoads, Leela Raghava Jaidev Chakka, Samrat Choudhury, Mohammed Maniruzzaman

**Affiliations:** 1 Pharmaceutical Engineering and 3D Printing (PharmE3D) Lab, School of Pharmacy, University of Mississippi, University, MS 38677 USA; 2 School of Engineering, University of Mississippi, University, MS 38677 USA

**Keywords:** Artificial intelligence, Machine learning, 3D printing, Fused deposition modeling, Polylactic acid, Polyvinyl alcohol, Thermoplastic urethane, And optimization

## Abstract

Three-dimensional printing (3DP) holds significant potential for developing personalized pharmaceutical oral dosage forms (printlets). 3D printing has the advantage of fabricating complex geometric structures for versatile drug release profiles, enhancing patient preference, palatability and swallowability, reducing the pill burden, and increasing dose accuracy. Optimizing printing parameters is crucial in determining the quality of the printlets during dosage development. The integration of machine learning (ML) can reduce production costs and time through parameter optimization based on trained datasets. This research is focused on optimizing parameters for fused deposition modeling (FDM) based batch and continuous printing methods. The algorithm was trained using a three-level full factorial design, which generated data in the form of printlets with different parameters. Both defect and defect-free printlets were analyzed using image segmentation. Machine learning tools including Gaussian Process Regressor (GPR) and Efficient Global Optimization (EGO) were used to predict and select processing parameters for a targeted percentage surface defect. The final trained algorithm predicted new parameter sets for both batch (R^2^-0.8783) and continuous (R^2^-0.9364) printing methods to achieve zero defects, and the same was confirmed through printing and characterization of printlets which showed no defects. The algorithm was later adapted successfully to a variety of materials within the temperature range of 190–220 ℃ and predicted zero-defect printlets. Scanning electron microscopy (SEM) revealed the absence of defects on the surfaces of the materials. Results showed that flow rate (110 and 120 mm^3^/s) had a significant impact on printlet quality withwithout defects for both batch and continuous printing, compared to print speed, print temperature, and infill density. This research provides new insights into the development of optimized FDM printlets using batch and continuous printing with adaptive machine learning for pharmaceutical dosage manufacturing.

**Supplementary Information:**

The online version contains supplementary material available at 10.1007/s13346-025-02006-4.

## Graphical abstract

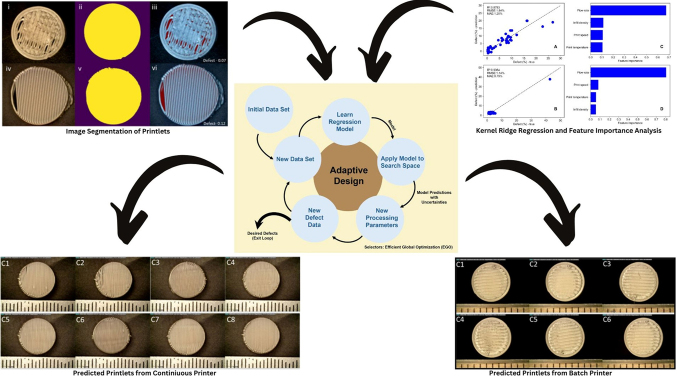

## Introduction

Additive manufacturing (AM) is a promising 3DP technology in the pharmaceutical field for the development of personalized drug delivery and medical devices in a cost-effective manner compared to conventional techniques [1]. The 3DP method of additive manufacturing deposits material in a layer-by-layer fashion. This technology has been widely accepted and explored ever since a research paper was published in the drug delivery sector in 1996 by Benjamin M. Wu et al. discussing the merits and possibilities of 3D printed combinations [2]. The advantages of employing 3D printing technology are manufacturing dosage forms in small batches, tailoring personalized dosages, optimizing shapes, sizes, and release characteristics to suit the patient’s needs [3, 4]. A recent advancement in this area is the commercial tablet, Spritam^®^ (Levetiracetam), developed by Aprecia Pharmaceuticals using their ZipDose^®^ 3D printing technology. The U.S. Food and Drug Administration (USFDA) has approved this as the first 3D-printed orally disintegrating dosage form [5]. The dosage form is designed digitally using Computer-aided design (CAD), which allows the researchers to develop complex designs. Fused deposition modeling (FDM) is one of the most prevalent technologies that use thermoplastic filaments extruded through Hot Melt Extrusion (HME) [6]. The FDM printer comprises a feeding gear that pushes the filament into the printer head, which includes of a heater (which heats up to 250 ℃) and a nozzle that lays the melted polymer filament in a layer-by-layer manner.

Currently, FDM is compatible with a diverse selection of materials, including PLA (Polylactic acid), PETG (Polyethylene terephthalate glycol), Flex, Nylon, ABS (Acrylonitrile butadiene styrene), PVA (Polyvinyl alcohol), PC (Polycarbonate), and PP (Polypropylene). PLA, known for its biodegradability and biocompatibility properties, is the most widely used material for filament fabrication in biomedical applications [7]. PLA has been widely used in tissue engineering, drug delivery, orthopedic devices, and implants because of its biodegradable and biocompatible nature [8]. Therefore, we used the PLA filament as a model filament to optimize the print parameters. In this research, a FDM printer with fixed print surface area (XY is 250 * 220 mm) and a nozzle at a 90-degree angle to the build surface was referred to as a batch printer or batch printing method. A conveyor build surface with an infinite z-axis (XY is 200 * infinity) and a nozzle oriented at 45-degree angle to the build surface was referred to as a continuous printer or continuous printing method for the fabrication of the dosage forms (PLA 3D structures). Our previous research demonstrated that the conveyor belt can reduce and eliminate the need for the utilization of support structures during the printing process [9].

In 3D printing, the most crucial stage to achieve a good quality printlet is the optimization of printing parameters. The quality of the printlet is influenced by a significantly large number of parameters related to the operation (e.g. flow rate, print speed, print temperature, wall thickness, layer height, top thickness, bottom thickness, and infill percentage, and infill pattern.) [10], the machine (e.g. nozzle diameter, raster angle, print orientation, raster width) [11], and the material/ink (e.g. density, viscosity, and polymer particle size etc.) [12, 13]. The integration of AM with machine learning (ML) can help optimize the combination of print parameters by reducing the time and cost of achieving high-quality printlets [14, 15]. Previous studies conducted the optimization of FDM parameters to reduce the printing time, surface roughness, volume percentage error, and critical factors that affect the quality of 3D printed objects. The self-learning model yielded optimal results by minimizing Mean Squared Error (MSE) and achieving large regression values (R_L_). Nozzle temperature, layer thickness, printing speed, and raster width were considered [16].

Ferretti et al. studied the optimization of printing parameters for reducing low-level defects using PLA. Mathematical modeling was used to reduce the defect volume by modifying the parameters such as layer dimensions, the overall number of shells, the extrusion multiplier, and temperature, which are filament-specific printing parameters. It was found that parameters such as nozzle diameter, extrusion width, and layer height can lead to a reduction in defect volumes. The defects were no longer seen once it was completely optimized [17]. There are other reports of the application of ML tools in combination with experiments and modeling to explore the FDM process. For example, ML tools such as Random Forest, Bayes, support vector machines, and artificial neural networks were utilized to establish a linkage between the processing parameters and surface roughness [16–20] in FDM printed parts of PLA and polyvinyl butyral. Similarly, a nonlinear regressor was used to predict the FDM processing parameters needed for a desired set of mechanical properties [21, 22] such as tensile strength, impact strength, and flexural strength of printed PLA samples. Besides the properties of the printed samples, the FDM printing process has also been investigated via ML. For example, Cohan’s group performed an ensemble-based Random Forest ML model to predict the deposition angle of the FDM process [23]. Overall, only a limited number of reported literatures have identified the operating parameters for a targeted FDM printing behavior. Navigating the vast combinatorial search space of operating parameters for a targeted FDM printing behavior is a non-trivial, time-consuming, and costly process. However, relatively less effort has been dedicated toward applying data science tools at the data generation stage, which can potentially further accelerate process optimization.

We applied our adaptive design approach to optimize processing parameters for a targeted fraction of surface defects during FDM printing. The study tested PLA, PVA, and TPU materials, chosen for their lower extrusion temperatures (190–220℃). Printlets were fabricated using predicted defect-free parameters for both batch and continuous printers. The dimensions were measured, and the model’s performance was statistically analyzed using a single-factor ANOVA with different material combinations for both printing methods. The goal was to optimize the 3D printing machine parameters using ML models rather than incorporating drugs. An initial dataset of PLA printlets with varying defects under different printing conditions (flow rate, infill density, print temperature, and print speed) referred to as combinations (C) was used as the training set. The model was trained on the training dataset, which predicted parameter sets for 0 and 15% defects (batch) and 0 and 30% defects (continuous). The predictions were then experimentally validated.

## Materials and methods

### Materials

PLA filament was purchased from Prusa Research (Prusament, Prusa Research, Czech Republic) while PVA and TPU Build Series filaments were purchased from Matter Hackers (Matter Hackers, California, U.S.A). The filaments, each with a diameter of 1.75 mm, were used as received without any further modifications.

### Design of experiments (DoE)

A DoE with full factorial design was performed to gather data in a systematic method and effectively explore the relationships between input factors (parameters) and output response (defects). Three-level full-factorial designs were utilized to obtain 81 (Table S1) and 83 (Table S2) combinations of four parameters (print speed, infill density, print temperature, and flow rate) for batch and continuous printer, respectively. These four parameters were varied to gather different sets of combinations while wall thickness, layer height, top thickness, and bottom thickness were kept constant throughout the study. Table S1 and S2 are available in the supplementary information.

### Design of printlet and 3D printing

The computer-aided design of the printlet was carried out using Tinkercad (Autodesk, U.S.A.). A cylindrical shape was adopted for the printlet with a diameter and height of 10 and 5 mm. The designed printlet was sliced using Prusa Slicer (v2.7.3, Prusa Research, Czech Republic) for batch printing, while Creality Slicer (v4.8.2, Shenzhen Creality 3D Technology, China) was used for the continuous printer. A zigzag pattern was selected for the infill, and a print head of 0.4 mm diameter was used with a bed temperature of 60 ℃, as common for both batch and continuous printing methods. Table 1 provides the processing parameters used in the fabrication of printlets. For each combination, five printlets were printed.

**Table 1:** Processing parameters for 3D printed printlets

Print Speed (mm/s)	Infill Density (%)	Print Temperature (℃)	Flow Rate (mm^3^/s)	Wall Thickness (mm)	Layer Height (mm)	Top Thickness (mm)	Bottom Thickness (mm)
10	0	180	80	0.4	0.2	0.4	0.4
30	20	200	100	0.4	0.2	0.4	0.4
50	40	220	120	0.4	0.2	0.4	0.4

### Characterization

A digital vernier caliper (SkillTech, USA) was used to measure the accuracy and reproducibility of the dimensions of the printed printlets from both printers. An optical microscope (Dino-Lite, AnMo Electronics Corporation, Torrance, CA, USA) was utilized to capture images of the printlets. The physical dimensions and images were further shared to perform image segmentation. The samples (*n* = 5) were characterized for further analysis.

### ML and adaptive design

#### Data collection

An initial dataset was generated comprising of percentage surface defects as a function of four printing parameters (flow rate, infill density, print speed, and print temperature), also known as features in our ML models. For each combination of printing parameters, printing was repeated 5 times. The batch and continuous printing dataset consisted of 81 and 83 different combinations of printing parameters, resulting in a total of 405 and 415 samples, respectively. In ML, each dataset was split into training and test sets in a ratio of 80:20 for model training and model performance evaluation, respectively.

#### Image segmentation of 3D printed dosage forms

**Fig. 1:**
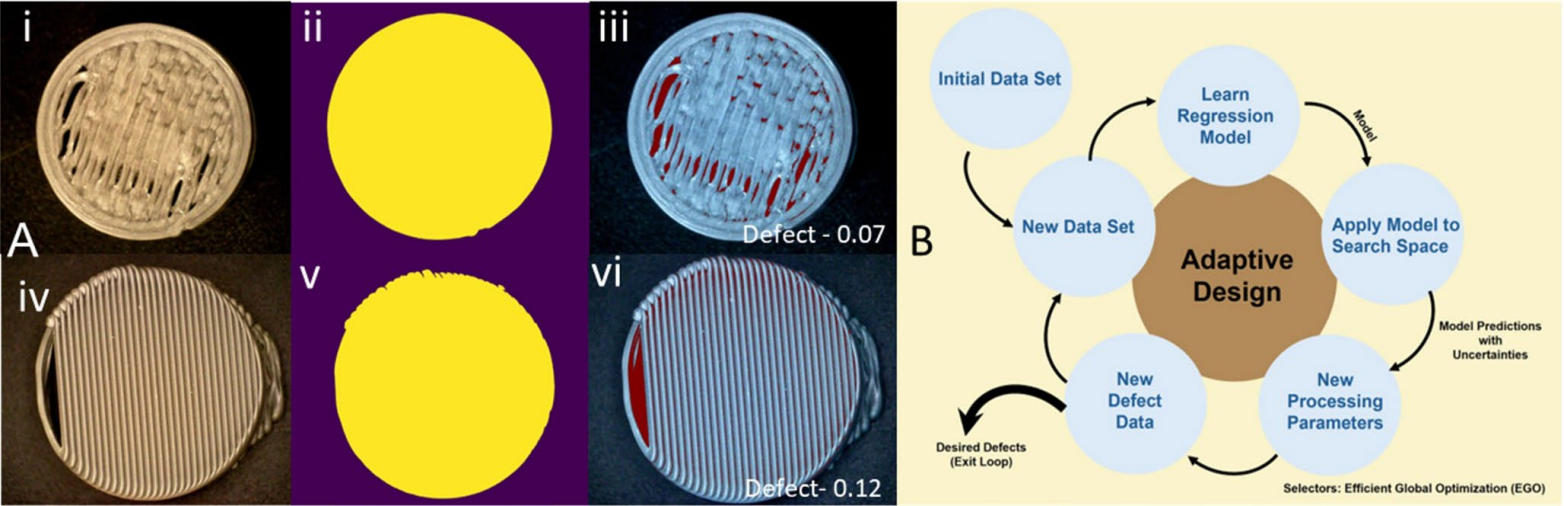
[**A**] Example of defect calculation process of a 3D printed dosage forms: [i] Original photograph of printlet from batch printer; [ii] Segmentation of printlet (yellow) from background (purple); [iii] Shaded regions of printlet (blue) and voids (red) for a grayscale threshold of 0.45, yielding a defect of 7%, [iv] Original photograph of printlet from continuous printer ; [v] Segmentation of printlet (yellow) from background (purple); [vi] Shaded regions of printlet (blue) and voids (red) for a grayscale threshold of 0.35, yielding a defect of 12%; [**B**] Adaptive design approach used in this study

The quantification of the percentage defects of 3D-printed printlets was performed using image segmentation methods. First, the image was converted to grayscale, and OpenCV (part of Python) was applied to find the threshold value, which separated the pixel values of the background from the boundary of the printlet. The backgrounds of the images were manually cropped for all images that were not accurately segmented using this approach. Next, a threshold was determined which precisely distinguished between the grayscale values of the pixels of the voids and the pixels of the 3D-printed printlet material. In the first dataset, a threshold of 0.45 was implemented for the batch printer while a threshold of 0.35 was implemented for the continuous printer. The thresholds were different between the two printers because printlets were printed with different textures, which reflected light differently during imaging. The total number of pixels in the printlet that were voids was divided by the total number of pixels in the printlet (voids and non-voids) to yield the percentage surface defect. Figure 1A depicts the process of image segmentation for batch and continuous printers while 1B depicts the adaptive design.

#### Data scaling

The four input features (print speed, infill density, print temperature, and flow rate) are recorded in different ranges and units (mm/s, %, °C, mm^3^/s, respectively), which may affect the accuracy of ML models due to the distance calculation rule of two points in training space. Many ML algorithms use the Euclidean distance to estimate the point-to-point distance, then data scaling can prevent any single feature to dominate the distance calculation. In this work, each feature data in the training set was scaled by standardization:

1 $${x_{new}} = \frac{{x - \mu }}{\sigma },$$

where ${x}_{new}$ is the data after scaling; $x$ is the original data; $\mu$ is the mean value of the feature in the original data; and $\sigma$ is the standard deviation of the feature. Since the test set works as an unknown space for model performance evaluation, the mean value ($\mu)$ and standard deviation ($\sigma$) for scaling the test set are identical to the values from the training set.

#### Kernel ridge regression (KRR)

*KRR* was used to predict the percentage of surface defects for a new set of features. *KRR* utilizes the kernel function to describe non-linear interactions between the features and the percentage of surface defects in a higher dimensional space compared to the original space [24–26]. To begin the training the initial input dataset is defined as: {*x*_*i*_, *y*_*i*_}, where *i* = 1, 2, …, *n*. *n* is the number of samples; *x*_*i*_ is the feature vector for the *i*th sample and *y*_*i*_ is the corresponding experimentally measured surface defect (or target) of the sample. In KRR, the predicted target is formulated as:

2 $${\hat y_j} = \sum\limits_{i = 1}^n {{w_i}K\left( {{x_i},{x_j}} \right)} ,$$

where ${\widehat{y}}_{j}$ is the *j*th predicted percentage surface defect; ${w}_{i}$ are the weights to be optimized; $K\left( {{x_i},{x_j}} \right)$ is the kernel function presenting a set of pairwise similarity comparisons between the samples [24, 27, 28]. In this work we used a polynomial kernel with a degree of 3 and coefficient of 0.2. The loss function to optimize the model is shown as:

3 $${\mathrm{Loss}} = \sum\limits_{i = 1}^n {{{({{\hat y}_i} - {y_i})}^2} + \lambda {w^T}Kw} ,$$

where ${\widehat{y}}_{i}$ is the *i*th predicted percentage defect; ${y}_{i}$ is the *i*th experimentally observed percentage defect; $\lambda$ is the regularization strength; $w$ is the weight vector of the function; $K$ is the kernel matrix $K\left( {{x_i},{x_j}} \right)$. The KRR model is developed by regularization strength of 0.5 and 0.7, respectively for batch and continuous process. The first term in Eq. (3) is to calculate the errors between the experimental defect and the predicted defect from the KRR model, while the second term is a penalty of large weight to avoid the overfitting issue. The expression for $w$ is formed by solving both Eqs. (2) and (3):

4 $$w = {(K + \lambda I)^{ - 1}}y,$$

where $K$ is the kernel matrix; and is the identity matrix. K-fold cross-validation (K = 10), and grid search were applied to estimate the hyperparameters of the *KRR* model to avoid overfitting issues.

#### Random forest

Random forest was utilized to investigate the relative importance of the four features in governing the percentage of surface defects. This method is more flexible in describing the nonlinear relationships between the combinations and the target numerically, and confronting the overfitting issue compared to other commonly used feature importance techniques (F-value, Mutual Information, Pearson’s correlation coefficient, etc.) [29, 30]. At each node for a given decision tree of Random Forest, feature importance is evaluated as:

5 $$\displaylines{{F_{jk}} = {W_k}{C_k} - {W_{left\left( k \right)}}{C_{left\left( k \right)}} \cr - {W_{right\left( k \right)}}{C_{right\left( k \right)}}, \cr} $$

where ${F_{jk}}$ is the importance of node *k* for feature *j*; ${W_k}$ is the weighted number of samples reaching node *k*; ${C_k}$ is the impurity value of node *k* (calculated by MSE); ${left(k)}$ is child node from left split on node *k*; $right(k)$ is child node from right split on node *k*. Then for feature importance in each decision tree:

6 $${F_j} = \frac{{\sum\nolimits_{_{k:node\:k\:splits\:on\:feature\:j}} {{F_{jk}}} }}{{\sum\nolimits_{_{h \in all\:nodes}} {{F_{jh}}} }},$$

where ${F_j}$ is the importance of feature *j*; ${F_{jk}}$ is the importance of node *k* for feature *j*; ${F_{jh}}$ is the importance of node *h* for feature *j*. The final importance for each feature is the average importance across all the decision trees.

The hyperparameters of each Random Forest model were optimized by grid search as listed below. The trained Random Forest for batch printing had 400 trees, 160 as the maximum depth of the decision tree, 2 features to apply at the node for the best split, 4 as the minimum number of samples to split an internal node, and 1 as the minimum number of samples at the leaf node. For continuous printing, the trained Random Forest was composed of 2,000 trees, a maximum depth of 50, 4 features applied at the node for the best split, a minimum of 12 samples to split an internal node, and a minimum of 3 samples at the leaf node.

#### Adaptive design

Previous materials informatics works on the FDM process focus on collecting experimental or computational samples and recognizing the pattern among the samples to optimize the processing conditions using common ML classifiers and regressors. However, these models possibly targeted local optimal due to the uncertainty of predictions in the big search space composed of multiple features as well as the sample size limited by experiments and modeling. In this paper, adaptive design, which provides the uncertainty of predictions, is used to guide the experiment to synthesize the printlet with optimal surface performance [31–33].

The operating principle of adaptive design is described in Fig. 1B [33]. The initial dataset is used to train the Gaussian regression models. Gaussian Process regressor follows an assumption function defined by a mean function and a covariance function. In the Gaussian Process regressor, it’s assumed that:

7 $$y = f\left( x \right) + \mathcal{N}\left( {0,{\sigma ^2}} \right),$$

where $f\left(x\right)$ is the function to be estimated, and $\mathcal{N}\left(0,{\sigma}^{2}\right)$ denotes a normal distribution with a mean value of 0 as well as a standard deviation of $\sigma$ to describe the error. Unlike other regressors (linear regressor, kernel ridge regressor, etc.), $f\left(x\right)$ is a set of functions in an infinite-dimensional Gaussian distribution. In this study, the mean function is set to be 0. The covariance is a square exponential function:

8 $$\displaylines{{\mathrm{cov}}[f\left( x \right),f\left( {x')} \right] = \cr {\mathrm{exp}}\left( { - \theta {{\left\| {x - x'} \right\|}^2}} \right) + \delta \left( {x,x'} \right){\sigma _n}, \cr} $$

where ${\theta}$ and ${\sigma}_{n}$ are free parameters generated by maximum likelihood and cross-validation. The search space can be applied to the trained Gaussian Process regression model to calculate the mean and standard deviation for each candidate from the search space. Mean and standard deviation work as predicted defect and uncertainty for each candidate in the selector [28].

The selector, Efficient Global Optimization determines the next set of processing parameters for the experiment following the Expected Improvement (EI) function ($E\left(x\right))$ [34]:

9 $$\displaylines{ E\left( x \right) = \left( {{\mu ^*} - \mu \left( x \right)} \right) \cr \Phi \left( {\frac{{{\mu ^*} - \mu \left( x \right)}}{{\sigma \left( x \right)}}} \right) + \sigma \left( x \right)\phi \left( {\frac{{{\mu ^*} - \mu \left( x \right)}}{{\sigma \left( x \right)}}} \right), \cr} $$

where ${\mu}^{*}$ is the value of the best-measured material; $\mu\left(x\right)$ and $\sigma\left(x\right)$ are the mean and standard deviation of the candidate; $\Phi$ and $\phi$ are the cumulative and probability density functions of $\mathcal{N}\left( {0,1} \right)$, respectively. Then the next potential sample selected is:    

10 $${x_{next}} = {\mathrm{arg}}\mathop {{\mathrm{max}}}\limits_x \left( {E\left( x \right)} \right),$$

Hence, in adaptive design under this study, there are two extreme cases: if all the candidates in the search space have a standard deviation of 0, the candidate with the best-predicted defect will be selected (exploitation); if there are candidates with high standard deviation, they will be the choice for next set of experiments (exploration). In the intermediate case, a trade-off between exploration (global search) and exploitation (local search) is balanced in suggesting the next experiments. If the synthesized sample demonstrates the desired percentage of surface defect, this set of processing parameters will be output, and the loop ends. Otherwise, the synthesized sample will augment the current dataset, and the loop will continue until the targeted percentage defect is attained.

#### Evaluation metrics

Three metrics were used in this work to score the ML model performance: coefficient of determination (R^2^), mean absolute error (MAE), and root mean square error (RMSE). Among these metrics, R^2^ tests how strongly the test data fits the trained model; MAE calculates the average of the residuals; RMSE presents the variance of the residuals. The formula for expressing each metric is shown below:

11 $${{\mathrm{R}}^2} = 1 - \frac{{\sum\nolimits_{i = 1}^n {{{\left( {{y_{i - }}{{\hat y}_i}} \right)}^2}} }}{{\sum\nolimits_{i = 1}^n {{{\left( {{y_{i - }}{{\bar y}_i}} \right)}^2}} }},$$

12 $${\mathrm{MAE}} = \frac{1}{n}\sum\nolimits_{i = 1}^n {\left| {{{\hat y}_i} - {y_i}} \right|} ,$$

13 $${\mathrm{RMSE}} = \sqrt {\frac{1}{n}\sum\limits_{i = 1}^n {{{({{\hat y}_i} - {y_i})}^2}} } ,$$

where *n* is the total number of samples, ${\bar y_i}$ is the mean value of the percentage experimental defect; ${\widehat{y}}_{i}$ is the predicted percentage defect; and ${y}_{i}$ is the experimental percentage defect.

All the ML algorithms are implemented in Scikit-learn library.

#### Printing printlets using ML predicted parameters

The printing of printlets was performed to validate the ML predictions for both continuous and batch printing and compare them with the experimental results. The printlets with 0, 1, 10, and 15% defects for batch printers were printed, while 0, 1, and 30% defect printlets were printed for continuous printers. The printlets were further utilized for gathering physical dimensions using vernier calipers, capturing images (digital camera), and calculating the contact angle.

#### Measurement of contact angle

The printlets were kept on a surface and the water droplet was allowed to drip on the printlet. The image was captured using an optical microscope (Dino-Lite, AnMo Electronics Corporation, Torrance, CA, USA) and the contact angle was measured using Image J software (v1.54j, National Institute of Health and Laboratory for Optical and Computational Instrumentation, University of Wisconsin, USA). In addition to the contact angle, the mean, maximum, and minimum degrees were calculated as well. Contact angle measurements were conducted on 5 printlets each for both batch and continuous printers.

#### Scanning electron microscopy

Scanning electron microscopy (SEM) was conducted to assess the surface morphologies of printlets for both batch and continuous printers using a JSM-7200FLV (JEOL, Peabody, MA, USA) with an accelerating voltage of 8 kV. Double-sided carbon tape was employed to affix all samples onto the SEM stubs. All samples were sputter-coated with platinum and maintained in an argon environment. The Denton Desk V TSC sputter coater (Denton Vacuum, Moorestown, NJ, USA) was employed to sputter-coat all samples before imaging [35].

#### Statistical analysis

A one-way Analysis of Variance (ANOVA) was conducted to evaluate the model’s efficiency across different materials and to determine any statistically significant differences between printlets produced using different printing methods. A significance threshold of *p < 0.05 was set for the analysis.

## Results

### Physical characterization

**Fig. 2:**
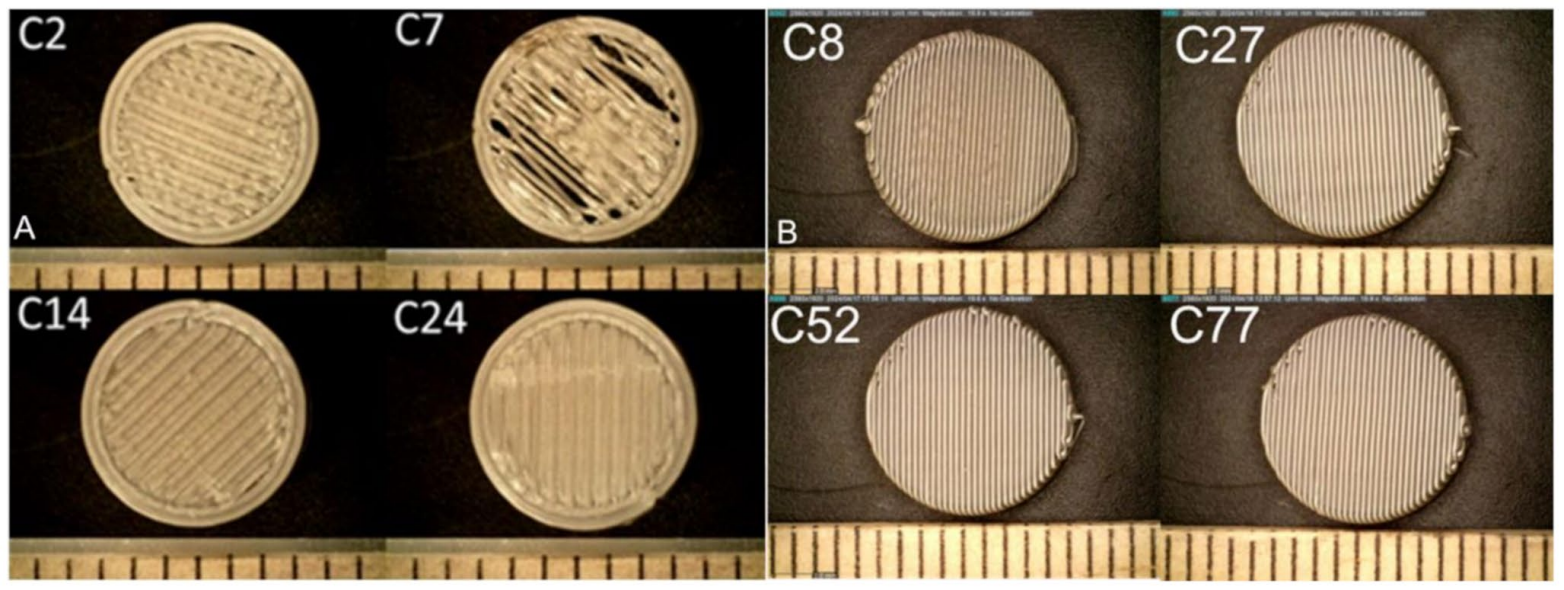
Printlets obtained using full factorial design for data generation [**A**] Batch printing [**B**] Continuous printing

Figures 2A&B depict a set of representative captured images for different combinations with varying flow rates, print temperatures, print speeds, and infill densities , printed using batch printer and continuous printer. The height and diameter were obtained for all combinations using the six-inch dial digital caliper. The printlets' height varied between 4.7 to 4.8 mm and the diameter between 9.8 to 10 mm for batch printing. The printlets printed using a continuous printer had a height with a range between 4.7 to 4.8 mg and a diameter of 9.9 to 10 mm. Combinations such as C2, C7, C14, etc. represent unique sets of parameters used for data generation. Detailed information about these parameters is provided in Tables S1 and S2 in the supplementary information.

### AI/ML

**Fig. 3:**
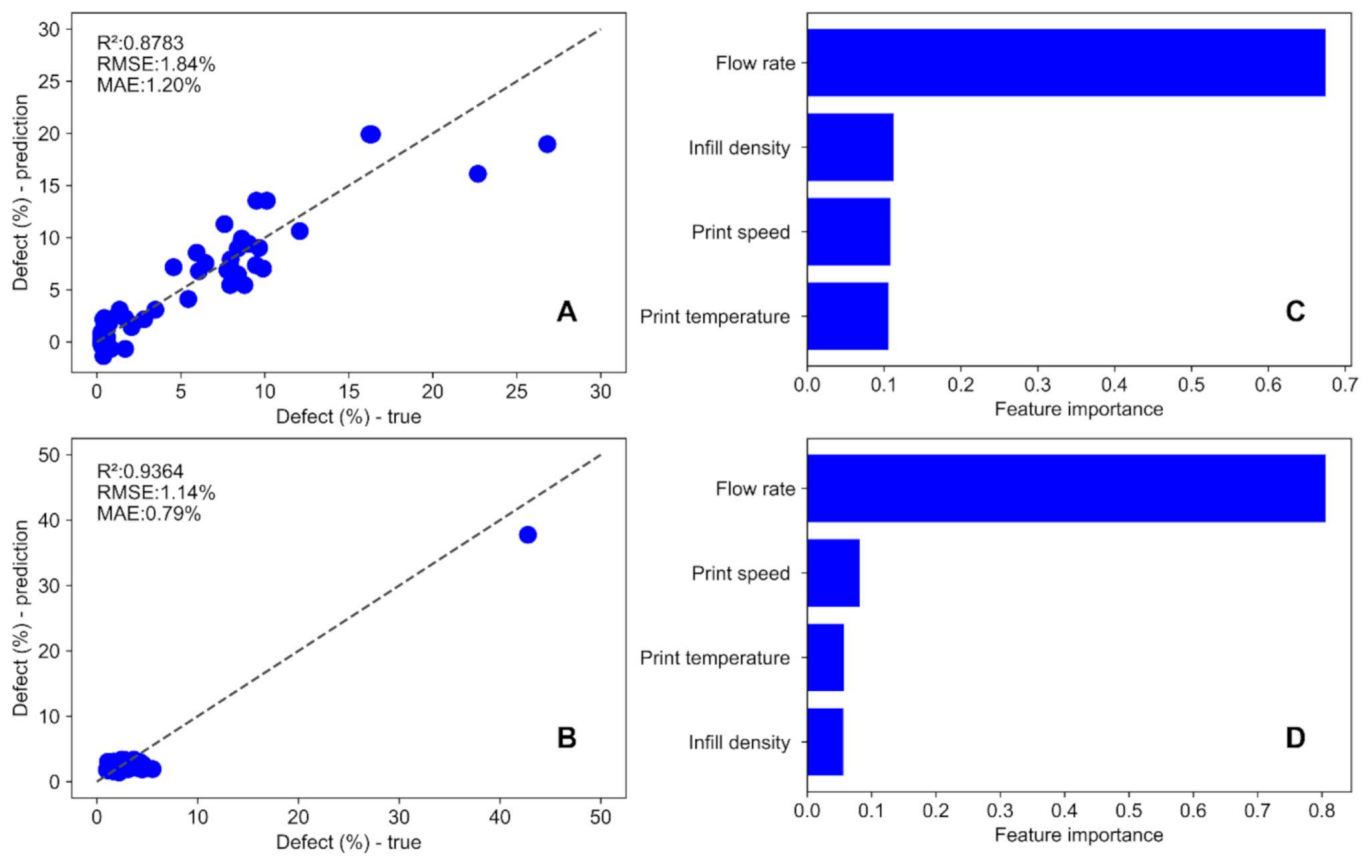
Kernel Ridge Regression [KRR] model performance using the dataset from printing [**A**] Batch Printer [**B**] Continuous Printer. Feature important analysis of each processing parameter using the dataset from printing [**C**] Batch Printer [**D**] Continuous Printer

Figure 3A exhibits the trained KRR model performance for the test set in the case of batch printing, where the x-axis represents the experimental percent defect and the y-axis is the percent KRR predicted defect. In this figure, the diagonal dashed line is the parity line where the experimental percent defect is equal to the percent predicted defect. Most of the data points are positioned around the parity line, which indicates that the four features can predict the percentage defect. The scores of this KRR model performance are R^2^: 0.8783, RMSE: 1.84%, and MAE: 1.20%. Although the overall R^2^ of the KRR model is less than 0.9, the data points less than 2% surface defects lie closely to the parity line (with an R^2^ = 0.9364). This indicates that the KRR model performs well for defects that are more relevant to drug delivery with little or no defects. Similarly, the trained and optimized KRR model for continuous printing is evaluated in Fig. 3B. In this figure, most data points are gathered in the vicinity of the parity line for x < 0.07 and y < 0.07. The metrics for the model (R^2^ of 0.9364, RMSE of 1.14%, and MAE of 0.79%) indicate the excellent capability of the KRR model to predict the percentage of defects.

The four features have an impact on the surface defects, so there may be a synergistic relationship among them in governing the formation of the defect. It is imperative to uncover this aspect to further understand the comprehensive relationship between the features and defects systematically. In Figs. 3C&D, the processing parameters are ranked by feature importance from high to low for batch and continuous printers, respectively. The horizontal axis is the feature importance, and the vertical axis is the list of the features. In Fig. 3C, except for the flow rate, the other 3 features, infill density, print speed, and print temperature have feature importance of similar magnitude. Infill density, print speed, and print temperature are much less important than flow rate in governing surface defects, but the sum of their feature importance is 0.33 in Fig. 3C and 0.18 in Fig. 3D, which is still appreciable compared to the feature importance of flow rate. Therefore, all four features are preserved for subsequent optimization of processing parameters using adaptative design approach.

### Optimization of 3D printed printlets using adaptive design strategy for batch printer

Tables 2 and 3 present results obtained from an adaptive design strategy for process optimization during batch printing with a desired percentage of the surface defect of 0% and 15%, respectively for PLA. As PVA and TPU share similar characteristics as PLA, similar processing parameters were used for PVA and TPU, and the resulting physical dimensions were listed. However, more robust ML models should include material features besides the four features related to processing conditions. Six sets of samples for each case, along with the probability (obtained from R^2^ of the trained GP model) that each candidate can lead to a targeted defect are also listed. For each ML formulation, 5 samples were synthesized, and the surface image for these samples was captured using an optical microscope and analyzed.

**Table 2:** Physical dimensions of 3D printed printlet using batch printer, 0% defects

Combinations	Print Speed (mm/s)	Infill Density (%)	Print Temperature (℃)	Flow Rate (mm^3^/s)	Probability	PLA		PVA		TPU	
						Height (mm)	Diameter (mm)	Height (mm)	Diameter (mm)	Height (mm)	Diameter (mm)
C1	50	20	200	110	0.7600	4.81	9.99	4.98 ± 0.01	9.75 ± 0.22	4.95 ± 0.03	9.48 ± 0.28
C2	20	20	180	110	0.7495	4.81	10	4.96 ± 0.04	9.89 ± 0.06	4.9 ± 0.04	9.71 ± 0.12
C3	10	30	220	110	0.6923	4.81	10	4.96 ± 0.03	9.96 ± 0.03	4.96 ± 0.05	9.86 ± 0.09
C4	50	10	190	120	0.6563	4.82	10	4.98 ± 0.01	9.84 ± 0.08	4.95 ± 0.02	9.7 ± 0.10
C5	30	30	200	110	0.5531	4.81	10	4.96 ± 0.03	9.84 ± 0.05	4.97 ± 0.03	9.81 ± 0.08
C6	40	10	190	110	0.5177	4.81	9.99	4.95 ± 0.06	9.81 ± 0.07	4.93 ± 0.02	9.48 ± 0.31

**Table 3:** Physical dimensions of 3D printed printlet using batch printer,15% defects

Combinations	Print Speed (mm/s)	Infill Density (%)	Print Temperature (℃)	Flow Rate (mm^3^/s)	Probability	Height (mm)	Diameter (mm)
C1	10	0	200	80	0.8696	4.8	9.99
C2	40	0	220	80	0.8271	4.8	9.99
C3	20	0	220	80	0.7502	4.8	9.99
C4	30	0	220	80	0.7134	4.8	9.99
C5	50	0	220	80	0.6851	4.8	9.99
C6	10	0	180	80	0.6465	4.8	9.99

**Fig. 4:**
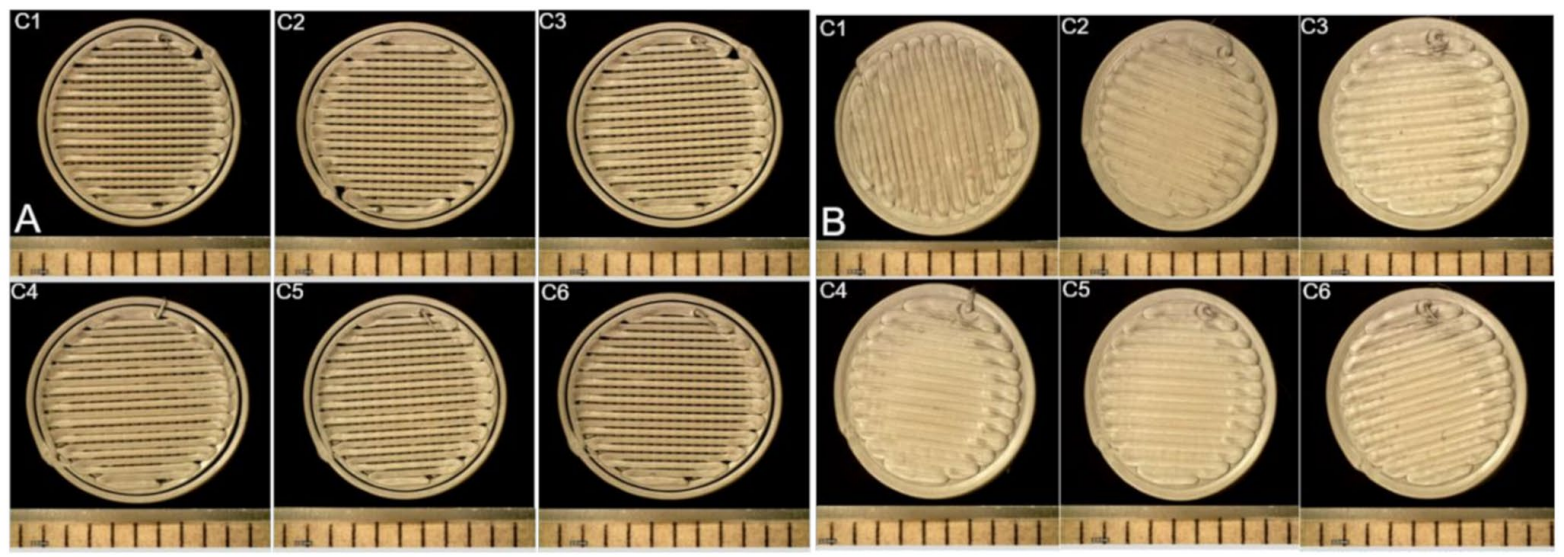
Predicted printlets printed using batch printer: [**A**] 15% defects [**B**] 0% defects

ML-guided experimental results are presented in Fig. 4A and B for 15% and 0%, respectively. While no defects were observed on the surface for all the ML-predicted processing conditions for 0% defect, obvious pores can be observed for all the ML-predicted processing conditions for 15% defect. Our quantitative image analysis shows that the average defect for 0% and 15% defects is 1.2% and 22.1%, respectively. Although our ML predicted processing conditions work well for 0% of targeted defects, the discrepancy between ML predictions and experimental measurements was observed for 15% of targeted defects. This is because the training data for batch printing had a limited number of samples with more than 10% defects, implying that there were not ample samples for the machine to learn the linkage between processing parameters (or features) and a higher percentage of surface defects. To further validate the potential of adaptive design in controlling the surface defects of the printed product, we further used our adaptive design strategy for targeted defects of 3% and 10% (see Supp. Tables S3 and S4 and Supp. Figure 1S in supplemental material). The predicted combinations were printed and were further evaluated for physical characterization and images. It was observed that our ML strategy works well for these two additional cases with surface defects less than or equal to 10%.

### Optimization of 3D printed printlets using adaptive design strategy for continuous printer

Tables 4 and 5 list eight and six sets of processing conditions obtained from an adaptive design approach for PLA with desired surface defects of 0% and 30% during continuous printing, respectively. In alignment with batch printing, ML-predicted processing conditions for PLA were used for PVA and TPU, and the corresponding physical dimensions are listed (Table 4). Comparable to batch printing, 5 samples were generated experimentally under each of the eight (0% defects) and six (30% defects) combinations.

**Table 4:** Physical dimensions of 3D printed printlet using continuous printer, 0% defects

Combinations	Print Speed (mm/s)	Infill Density (%)	Print Temperature (℃)	Flow Rate (mm^3^/s)	Probability	PLA		PVA		TPU	
						Height (mm)	Diameter (mm)	Height (mm)	Diameter (mm)	Height (mm)	Diameter (mm)
C1	30	5	210	75	0.8718	4.78	9.98	4.35 ± 0.05	9.64 ± 0.22	4.31 ± 0.11	9.37 ± 0.18
C2	50	40	220	65	0.8700	4.77	9.99	4.34 ± 0.06	9.76 ± 0.06	4.28 ± 0.06	9.55 ± 0.10
C3	30	15	220	90	0.8696	4.77	9.99	4.41 ± 0.08	9.90 ± 0.04	4.44 ± 0.06	9.53 ± 0.15
C4	40	40	220	80	0.7642	4.78	9.99	4.39 ± 0.02	9.91 ± 0.05	4.43 ± 0.05	9.74 ± 0.09
C5	40	25	210	90	0.6181	4.77	9.99	4.35 ± 0.08	9.92 ± 0.03	4.35 ± 0.09	9.76 ± 0.04
C6	45	25	215	95	0.5561	4.78	9.99	4.34 ± 0.09	9.89 ± 0.09	4.44 ± 0.03	9.73 ± 0.09
C7	15	15	220	120	0.8687	4.78	10	4.43 ± 0.04	9.97 ± 0.01	4.48 ± 0.01	9.73 ± 0.12
C8	50	5	205	80	0.8310	4.77	9.99	4.32 ± 0.08	9.84 ± 0.12	4.37 ± 0.13	9.39 ± 0.16

**Table 5:** Physical dimensions of 3D printed printlet using continuous printer, 30% defects

Combinations	Print Speed (mm/s)	Infill Density (%)	Print Temperature (℃)	Flow Rate (mm^3^/s)	Probability	Height (mm)	Diameter (mm)
C1	25	15	180	25	0.8592	4.77	9.97
C2	10	40	180	20	0.8373	4.57	9.77
C3	20	35	180	20	0.8186	4.57	9.76
C4	15	35	180	25	0.7081	4.77	9.98
C5	10	15	180	20	0.6944	3.75	9.97
C6	25	20	180	30	0.5965	4.78	9.97

**Fig. 5:**
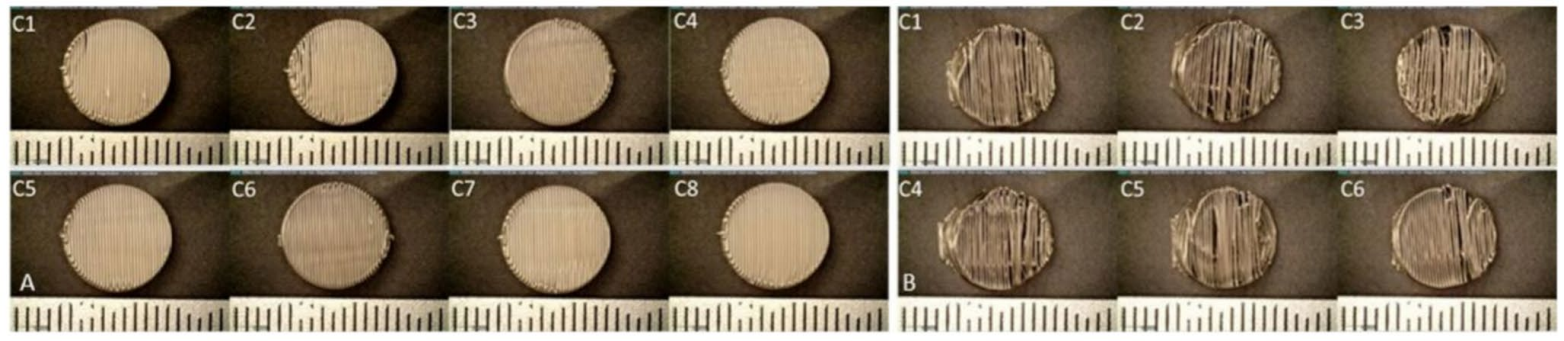
Predicted printlets printed using continuous printer [**A**] 0% Defects and [**B**] 30% defects

These samples were later synthesized, and the images of these samples were captured using an optical microscope and analyzed as presented in Fig. 5B (C1-C6). The experimental images (C1 & C2 in Fig. 5B) exhibit pores. This contrasts with ML predictions of 0% surface defects for samples C1 and C2 in Table 4. The reasons for the discrepancy can be explained based on the following observations. In Fig. 3D, we found that flow rate is the most important feature contributing around 80% toward the percentage of surface defect. The contribution of the flow rate is more than the contribution of the other three features combined. ML predicted combination (C1 & C2) in Table 4 both have flow rates under 80 mm^3^/s, while the initial dataset collected from continuous printing only includes a few samples with flow rates < 80 mm^3^/s. This implies that the machine did not have access to sufficient data to learn for cases with less than 80 mm^3^/s flow rate, leading to higher errors in ML predictions.

To confirm the viability of our ML approach, we synthesized two additional two ML-prediction combinations with high flow rates (see C7 and C8 in Table 4). The experimental results (Fig. 5A ) C7&C8 confirm that the two combinations lead to 0% surface defect. To generalize the capability of our ML framework, later we considered 30% surface defect as the next desired target during continuous printing, and the ML-predicted processing condition combinations were listed in Table 5. Compared to Fig. 5A, missing sections deteriorate the product quality in Fig. 5B. Given that for drug delivery surface defects close to 0% surface defects are of importance, an additional set of ML predictions for processing conditions needed for 1% surface is presented in Table S5 and Fig. 2S in the supplementary material. The experimental results validate ML predictions for the case with 1% surface defect.

### Measurement of contact angle for 3D printed printlets

**Fig. 6:**
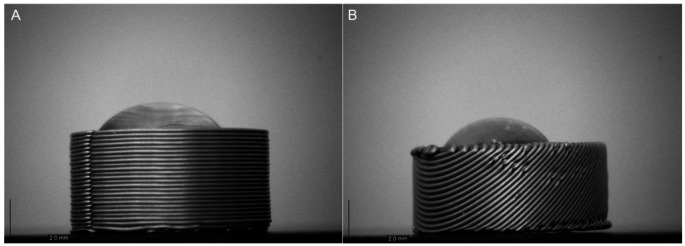
Representation of contact angle measurement [**A**] Batch printer [**B**] Continuous printer

The contact angle of 3D-printed printlets was calculated to measure their hydrophilic and hydrophobic nature. Largely, the contact angle depends on the type of material used and defects on the surface of the printlet . This was performed to validate the absence of defects on the surface of the printlet and to calculate its hydrophilicity and hydrophobicity. Figure 6 depicts the contact angles of printlets for batch and continuous printers. The contact angles for batch and continuous printers were calculated to be 45 ± 2 and 49 ± 1.9 degrees, respectively.

### Characterization

#### Scanning electron microscopy

**Fig. 7:**
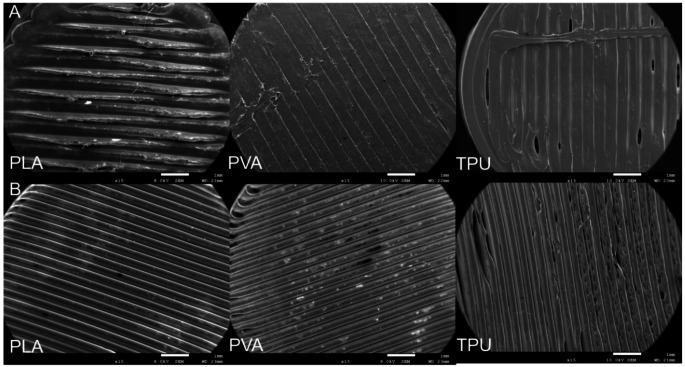
Scanning electron microscopic (SEM) images of predicted printlets from [**A**] Batch printer [**B**] Continuous Printer

The scanning electron microscopy (SEM) was performed to confirm the absence of defects on the surface of printlets that were printed using batch and continuous methods. Figure 7 depicts the microscopic images at a magnification of 15. The printlets that were printed with PLA and PVA had an absence of defects on the surface of the printlets for both batch and continuous printed printlets. The surface and edges of the printlets had an absence of defects which suggests that proper adhesion was achieved. The printlets that were printed with TPU suggested that the material had some defects on the surface.

**Fig. 8:**
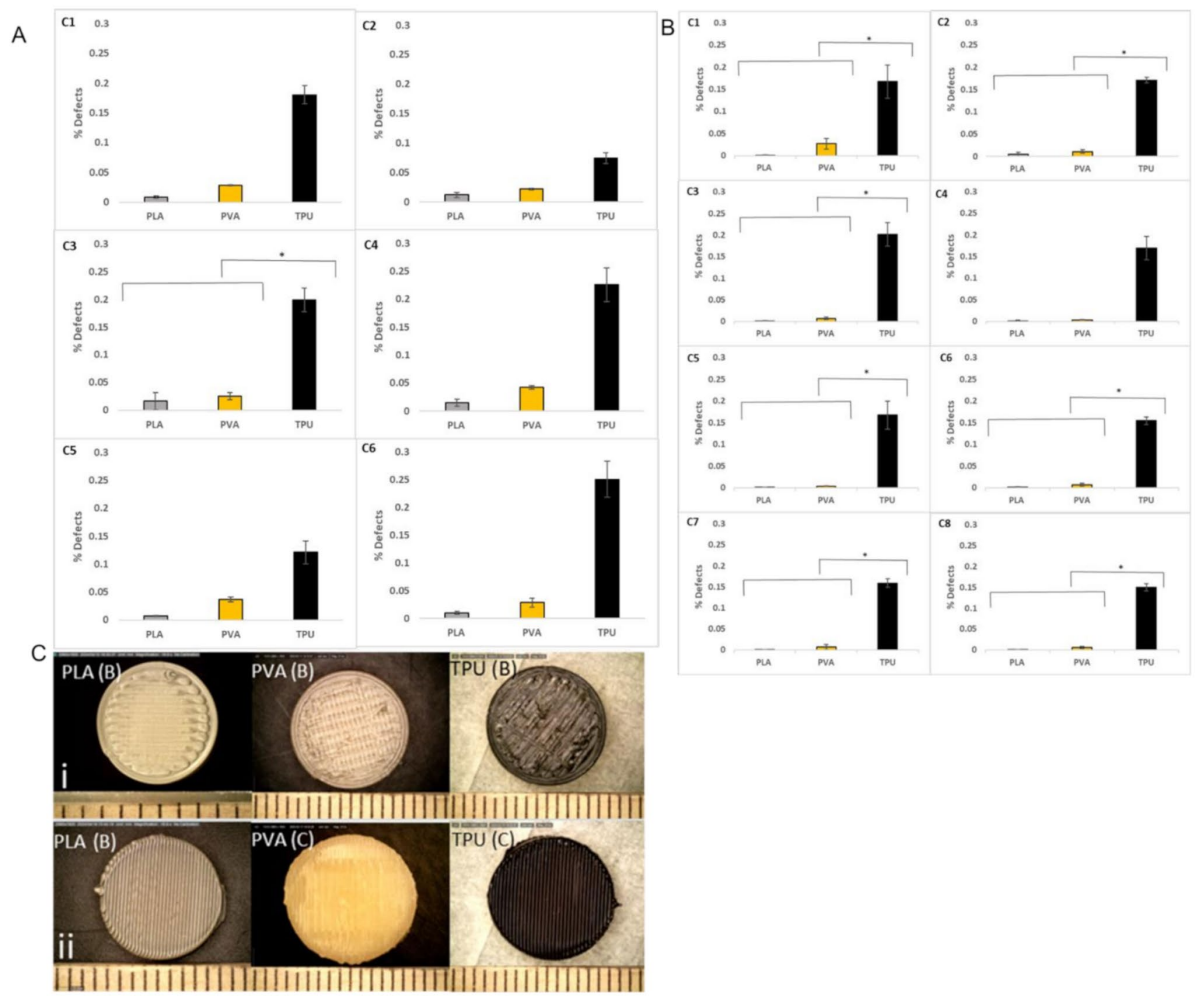
Comparison of materials and printlets from batch and continuous printers: [**A**] Comparison of materials plotted with the generated printing parameters for C1 to C6 combinations for batch printer. The data are represented by mean ± standard deviation for sample size *n* = 5. The statistical significance of *p* < 0.05 was achieved through one way ANOVA with post Turkey test for significance. [**B**] Comparison of materials plotted with the generated printing parameters for C1 to C8 combinations for continuous printer. The data are represented by mean ± standard deviation for sample size *n* = 5. The statistical significance of *p* < 0.05 was achieved through one way ANOVA with post Turkey test for significance. [**C**] Images of printlets printed with batch and continuous printer

Figures 8A&B present the statistical analysis conducted on printlets printed using both batch and continuous methods. The data obtained were statistically significant, with *p*-values less than 0.05 for all combinations of batch and continuous methods. Additionally, the defect values of printlets printed with PVA and TPU were significantly different from those printed with PLA. Figure 8C consists of printlets that were printed using PLA, PVA, and TPU with batch and continuous printers.

#### Mechanical strength

Texture analysis was conducted to evaluate the flexibility and mechanical strength of PLA, PVA, and TPU. The structures, printed using both batch and continuous printers under identical parameters (print speed, print temperature, flow rate, and infill density), were tested (Figure S3). The PLA structures fractured during testing for both printer types, while PVA and TPU structures exhibited elongation without breaking. It highlights the influence of material properties on mechanical performance—specifically, the rigidity of PLA leading to brittleness and the flexibility of PVA and TPU allowing them to deform under stress. These trends were consistent across both printer types, indicating that the type of printer did not significantly affect tensile behavior under the controlled conditions used.

As summarized in Table S6 the breaking force of structures varied with material types and printing methods. PLA structures printed with the batch printer withstood a force of 444.2 N before breaking, while the continuous printer versions tolerated a higher force of 528.48 N, though both ultimately failed. In contrast, PVA and TPU showed lower peak forces (batch: 71.99 N and 53.04 N; continuous: 64.02 N and 63.99 N) but did not fracture, instead demonstrating notable elongation, with PVA stretching more than TPU. Figure S4 presents the corresponding tensile analysis. The results indicate the structures printed with the continuous printer exhibited higher tensile strength compared to batch-printed structures for PLA and TPU. However, for PVA, the batch-printed structure demonstrated slightly better tensile performance compared to the one printed using the continuous printer. The results also support our conjecture that the inclusion of material feature(s) is an important input for future ML models aimed at predicting the mechanical robustness of drug-loaded 3D-printed tablets.

## Discussion

The use of AI in drug formulation is crucial for minimizing failures, reducing time and cost in research and development, and enhancing decision making in selecting appropriate excipients, administration routes, and dosage forms. 3DP is emerging as a powerful tool for personalized medicine. The complexity of tablet manufacturing requires precise control of multiple parameters, and AI enables efficient optimization to achieve desired drug product outcomes [36, 37]. The parameters, print temperature, flow rate, infill density, and print speed, were varied using a three-level full factorial design to generate datasets for both batch and continuous printers. Meanwhile, other parameters-layer height, wall thickness, top thickness, and bottom thickness-were kept constant. Key parameters such as drug release profiles, dosage strengths, geometry, printing conditions, and patient-specific factors (e.g., age, weight, and medical history) must be finely tuned to develop individually dosage forms [36]. Table 1 outlines the specific values used in the design of experiments (DOE) for both printing methods. To calculate defect values of the printlets, image segmentation was performed on all printlets − 405 form the batch printer and 415 from the continuous printer.

Figure 1A illustrates the image segmentation process used to quantify defect. Figure 1B presents the adaptive ML model developed for predicting optimal parameter combinations. Figure 2 shows the printlets fabricated using the DOE-generated parameter combinations. These printlets were subsequently subjected to image segmentation, and the resulting defect values were fed into the adaptive design ML model. The model then generated parameter predictions corresponding to specific defect levels: 0 and 15% for batch printing and 0 and 30% for continuous printing. Kernel Ridge Regression (KRR) analysis, Fig. 3A&B, for both printer types demonstrated that the predicted defect values closely aligned with the parity line, confirming the model’s strong predictive capability for surface defects in pharmaceutical printlets. Previous work by Hanjun Wei et al. explored combinations of FDM printing parameters-including deposition layer thickness, printing temperature, speed, and infill density-using a T-S fuzzy neural network model. They found that deposition layer thickness significantly influenced PLA fiber morphology, and increasing infill density reduced air gaps [38].

Featuring the key analysis observed from Fig. 3C&D it is understood that while all parameters influenced surface defects to some extent, flow rate had the most significant impact. This underscores its dominant role in determining printlet quality in both batch and continuous 3D printing approaches. The findings align with earlier research by Blonk et al. and Sharma et al., which demonstrated that inadequate material flow during 3D printing leads to poor layer deposition and structural defects [39, 40]. Especially, excessive flow rates cause over-extrusion and overfill defects, while low flow rates lead to under extrusion [41–43], resulting in underfill defects and large voids between layers [40].

The batch printing of PLA, PVA, and TPU printlets with 0% defects (Table 2) showed that the dimensions of the printlets – specifically height and diameter – had no significant standard deviation for PVA and TPU, indicating good dimensional accuracy. The parameters used to produce PLA printlets with 15% defects, where the standard deviation was also found to be zero (Table 3). Figure 4 shows printlets with 15% and 0% defects produced using the batch printer. Printlets fabricated at flow rates above 80 mm³/s were fully sealed, with no visible pores on their surfaces or edges, likely due to sufficient material flow enabling proper layer adhesion. In contrast, printlets printed at 80 mm³/s exhibited poorer quality, with visible defects caused by inadequate material extrusion. Therefore, higher flow rates produced superior, defect-free printlets, while lower rates led to poor structural integrity. Tables S3 and S4 (Supplementary) present data on flow rates between 110 and 120 mm³/s.

The printlets produced from the continuous printer had poor adhesion, which was visually observed on both the surface and edges of printlets printed with lower flow rates and infill densities. This poor adhesion contributed to increased defects and reduced print quality. Similar observations were made by Kaveh et al., who concluded that flow rate, feed rate, and nozzle temperature are critical determinants of print quality when printing with PLA [44]. Table 4 presents the parameters for printlets with 0% defects produced using the continuous printer. PLA printlets showed a negligible standard deviation, indicating high dimensional consistency. While PVA and TPU printlets exhibited some standard deviation, it was not significant. Table 5 shows the parameters used to produce PLA printlets with 30% defects, where the standard deviation was also found to be negligible. Figure 5A&B compare printlets with 30% and 0% defects printed using the continuous printer. The 30% defective printlets exhibited extremely poor layer adhesion and were structurally fragile, likely due to low flow rates during printing. These results are consistent with findings by Blok et al. and Sharma et al., who reported that inadequate material supply and improper filament orientation lead to interlayer pores and reduced tensile strength [39, 40].

The contact angle measurements of printlets were below 75°, indicating the hydrophilic nature of the material and suggesting a smooth, defect-free surface (Fig. 6) [45]. A low contact angle corresponds to a well-formed surface layer, while a significantly lower or negligible contact angle would suggest surface defects, allowing water to penetrate the printlet. Surface morphology was further evaluated using scanning electron microscopy (Fig. 7A&B). Printlets made from PLA and PVA exhibited smooth, uniform surfaces, indicating consistent material deposition and minimal irregularities. In contrast, TPU printlets showed more surface defects, especially at the edges. This could be attributed to TPU’s flexibility, which may cause under-extrusion or result in rough surface finishes during printing [46].

The robustness of the printing process and accuracy of the predicted parameters of the AI/ML were observed from the statistical analysis (Fig. 8A&B) which confirmed that differences between batch and continuous printing methods were statistically significant (*p* < 0.05), indicating that the observed variations were not by chance or random. Printlets made with PVA and TPU had significantly different defect values compared to those made with PLA. Notably, the AI model successfully predicted 0% defect outcomes for PLA and PVA, while TPU printlets showed minor defects, likely due to the material’s inherent properties. These findings highlight the model’s robustness and versatility in optimizing the printing process across different materials. The printlets were intact and well-formed from various materials using both printing methods, with no visible surface defects.

The advantage of printing a personalized dosage is the print time, which provides the final output of the amount of tablets produced in the least time. The data observed from Fig. 8C show that the print speed was found to significantly influence the overall printing time in both continuous and batch printing methods. The continuous printer required 2–6 min to print a tablet, while the batch printer required 2–4 min, depending on infill density. Batch-printed printlets consisted of 25 layers, while continuous-printed printlets consisted of 51 layers. Increased infill density led to longer printing times in both cases. These observations align with previous findings by Suteja, who also reported that infill density plays a key role in determining print duration [47].

Overall, the printlets made from PLA, PVA, and TPU were successfully fabricated using both batch and continuous printers. The continuous printer outperformed the batch printer, particularly at a flow rate of 80 mm³/s. While the batch printer produced poor-quality printlets at this flow rate, the continuous printer maintained good quality. In continuous printing, print speed emerged as the second most significant factor after flow rate, with infill density and temperature contributing similarly.

## Conclusion

We demonstrated the integration of AI with 3DP to generate novel processing combinations targeted at reducing surface defects in pharmaceutical tablets (printlets). The results showed that the integration of AI/ML can successfully predict a new set of parameters to develop printlets with zero defects. The model was successful in predicting printlets for different materials. The initial *proof-of-concept* study has given a wealth of information to perform additional research for developing drug-loaded formulations to minimize the time, resources, and cost required for optimizing printing parameters. This work marks a significant step toward developing an algorithm capable of optimizing 3D-printed dosage forms. The current work can be further extended using different polymers and incorporating an active pharmaceutical ingredient to evaluate the robustness of the AI/ML model and assess its ability to deliver consistent drug dosage forms in vitro.

## Supplementary Information

Below is the link to the electronic supplementary material.

Supplementary data associated with this article can be found in the Appendix A

## Data Availability

Data will be made available on the request with the corresponding authors.
