# Supplementary material for: Optimization of print parameters for batch and continuous manufacturing of three-dimensional (3D) printed dosage forms using artificial intelligence and machine learning
Source: Drug Deliv Transl Res. 2025 Nov 4;16(6):2073–88. doi: 10.1007/s13346-025-02006-4 (PMC13183753; doi:10.1007/s13346-025-02006-4)
Supplement: Supplementary file 1 — Supplementary data associated with this article can be found in the Appendix A [file 13346_2025_2006_MOESM1_ESM.docx]

**Appendix A**

**Supplementary Information**

**Optimization of print parameters for Batch and Continuous Manufacturing of three-dimensional (3D) Printed dosage forms using Artificial Intelligence and Machine Learning**

Kshitij Chitnis^1#^, Yizhou Lu^2#^, Benjamin Rhoads^2^, Jaidev Chakka^1^, Samrat Choudhury^2^* & Mohammed Maniruzzaman^1^*

*^1^Pharmaceutical Engineering and 3D printing Lab (PharmE3D), School of Pharmacy, University of Mississippi, University, MS 38677, USA.*

*^2^School of Engineering, University of Mississippi, University, MS 38677, USA.*

# Equal Contribution

**Materials and Methods**

**Design and 3D printing of Dog bone structure for texture analysis**

The computer aided design of the dog bone structure was carried out according to ASTM D-638. The designed structure was later sliced using Prusa Slicer (v2.7.3, Prusa Research, Czech Republic) for batch printing while Creality Slicer (v4.8.2, Shenzhen Creality 3D Technology, China) for the continuous printer. A zig-zag pattern infill pattern was selected, and a 0.4mm diameter print head was used. A bed temperature of 60℃ was maintained for both batch and continuous processes.

Dog bone structures with dimensions of 3.4 * 19.0 * 115.0 mm (Figure 1) were printed to evaluate their strength and flexibility, confirming whether the predicted parameters produced mechanically robust structures. Mechanical testing was performed using a TA- XT2i Texture Analyzer (Stable Micro Systems Ltd., Godalming, England) equipped with a TA – 96B – W tensile module. The test was conducted using the following parameters: pre – test speed – 1.50 mm/s; test speed – 2.00 mm/s; post-test – 10.00 mm/s; Distance – 15.00 mm. A pressure of 50 kg was applied and a trigger force of 1.000 N. Exponent version 6.1.5.0 software (Stable Micro Systems Ltd, Godalming, UK) was used to monitor and generate the force - distance graph. Each test was performed five times.

**Results**

Table S1: 81 combinations with different baseline parameters, achieved using full factorial design.

| Combinations | Print Speed (mm/s) | Infill Density (%) | Print Temperature (℃) | Flow Rate  (mm^3^/s ) | Wall thickness (mm) | Layer height (mm) | Top Thickness (mm) | Bottom Thickness (mm) |
| --- | --- | --- | --- | --- | --- | --- | --- | --- |
| 1 | 10 | 0 | 180 | 80 | 0.4 | 0.2 | 0.4 | 0.4 |
| 2 | 10 | 0 | 180 | 100 | 0.4 | 0.2 | 0.4 | 0.4 |
| 3 | 10 | 0 | 180 | 120 | 0.4 | 0.2 | 0.4 | 0.4 |
| 4 | 10 | 0 | 200 | 80 | 0.4 | 0.2 | 0.4 | 0.4 |
| 5 | 10 | 0 | 200 | 100 | 0.4 | 0.2 | 0.4 | 0.4 |
| 6 | 10 | 0 | 200 | 120 | 0.4 | 0.2 | 0.4 | 0.4 |
| 7 | 10 | 0 | 220 | 80 | 0.4 | 0.2 | 0.4 | 0.4 |
| 8 | 10 | 0 | 220 | 100 | 0.4 | 0.2 | 0.4 | 0.4 |
| 9 | 10 | 0 | 220 | 120 | 0.4 | 0.2 | 0.4 | 0.4 |
| 10 | 10 | 20 | 180 | 80 | 0.4 | 0.2 | 0.4 | 0.4 |
| 11 | 10 | 20 | 180 | 100 | 0.4 | 0.2 | 0.4 | 0.4 |
| 12 | 10 | 20 | 180 | 120 | 0.4 | 0.2 | 0.4 | 0.4 |
| 13 | 10 | 20 | 200 | 80 | 0.4 | 0.2 | 0.4 | 0.4 |
| 14 | 10 | 20 | 200 | 100 | 0.4 | 0.2 | 0.4 | 0.4 |
| 15 | 10 | 20 | 200 | 120 | 0.4 | 0.2 | 0.4 | 0.4 |
| 16 | 10 | 20 | 220 | 80 | 0.4 | 0.2 | 0.4 | 0.4 |
| 17 | 10 | 20 | 220 | 100 | 0.4 | 0.2 | 0.4 | 0.4 |
| 18 | 10 | 20 | 220 | 120 | 0.4 | 0.2 | 0.4 | 0.4 |
| 19 | 10 | 40 | 180 | 80 | 0.4 | 0.2 | 0.4 | 0.4 |
| 20 | 10 | 40 | 180 | 100 | 0.4 | 0.2 | 0.4 | 0.4 |
| 21 | 10 | 40 | 180 | 120 | 0.4 | 0.2 | 0.4 | 0.4 |
| 22 | 10 | 40 | 200 | 80 | 0.4 | 0.2 | 0.4 | 0.4 |
| 23 | 10 | 40 | 200 | 100 | 0.4 | 0.2 | 0.4 | 0.4 |
| 24 | 10 | 40 | 200 | 120 | 0.4 | 0.2 | 0.4 | 0.4 |
| 25 | 10 | 40 | 220 | 80 | 0.4 | 0.2 | 0.4 | 0.4 |
| 26 | 10 | 40 | 220 | 100 | 0.4 | 0.2 | 0.4 | 0.4 |
| 27 | 10 | 40 | 220 | 120 | 0.4 | 0.2 | 0.4 | 0.4 |
| 28 | 30 | 0 | 180 | 80 | 0.4 | 0.2 | 0.4 | 0.4 |
| 29 | 30 | 0 | 180 | 100 | 0.4 | 0.2 | 0.4 | 0.4 |
| 30 | 30 | 0 | 180 | 120 | 0.4 | 0.2 | 0.4 | 0.4 |
| 31 | 30 | 0 | 200 | 80 | 0.4 | 0.2 | 0.4 | 0.4 |
| 32 | 30 | 0 | 200 | 100 | 0.4 | 0.2 | 0.4 | 0.4 |
| 33 | 30 | 0 | 200 | 120 | 0.4 | 0.2 | 0.4 | 0.4 |
| 34 | 30 | 0 | 220 | 80 | 0.4 | 0.2 | 0.4 | 0.4 |
| 35 | 30 | 0 | 220 | 100 | 0.4 | 0.2 | 0.4 | 0.4 |
| 36 | 30 | 0 | 220 | 120 | 0.4 | 0.2 | 0.4 | 0.4 |
| 37 | 30 | 20 | 180 | 80 | 0.4 | 0.2 | 0.4 | 0.4 |
| 38 | 30 | 20 | 180 | 100 | 0.4 | 0.2 | 0.4 | 0.4 |
| 39 | 30 | 20 | 180 | 120 | 0.4 | 0.2 | 0.4 | 0.4 |
| 40 | 30 | 20 | 200 | 80 | 0.4 | 0.2 | 0.4 | 0.4 |
| 41 | 30 | 20 | 200 | 100 | 0.4 | 0.2 | 0.4 | 0.4 |
| 42 | 30 | 20 | 200 | 120 | 0.4 | 0.2 | 0.4 | 0.4 |
| 43 | 30 | 20 | 220 | 80 | 0.4 | 0.2 | 0.4 | 0.4 |
| 44 | 30 | 20 | 220 | 100 | 0.4 | 0.2 | 0.4 | 0.4 |
| 45 | 30 | 20 | 220 | 120 | 0.4 | 0.2 | 0.4 | 0.4 |
| 46 | 30 | 40 | 180 | 80 | 0.4 | 0.2 | 0.4 | 0.4 |
| 47 | 30 | 40 | 180 | 100 | 0.4 | 0.2 | 0.4 | 0.4 |
| 48 | 30 | 40 | 180 | 120 | 0.4 | 0.2 | 0.4 | 0.4 |
| 49 | 30 | 40 | 200 | 80 | 0.4 | 0.2 | 0.4 | 0.4 |
| 50 | 30 | 40 | 200 | 100 | 0.4 | 0.2 | 0.4 | 0.4 |
| 51 | 30 | 40 | 200 | 120 | 0.4 | 0.2 | 0.4 | 0.4 |
| 52 | 30 | 40 | 220 | 80 | 0.4 | 0.2 | 0.4 | 0.4 |
| 53 | 30 | 40 | 220 | 100 | 0.4 | 0.2 | 0.4 | 0.4 |
| 54 | 30 | 40 | 220 | 120 | 0.4 | 0.2 | 0.4 | 0.4 |
| 55 | 50 | 0 | 180 | 80 | 0.4 | 0.2 | 0.4 | 0.4 |
| 56 | 50 | 0 | 180 | 100 | 0.4 | 0.2 | 0.4 | 0.4 |
| 57 | 50 | 0 | 180 | 120 | 0.4 | 0.2 | 0.4 | 0.4 |
| 58 | 50 | 0 | 200 | 80 | 0.4 | 0.2 | 0.4 | 0.4 |
| 59 | 50 | 0 | 200 | 100 | 0.4 | 0.2 | 0.4 | 0.4 |
| 60 | 50 | 0 | 200 | 120 | 0.4 | 0.2 | 0.4 | 0.4 |
| 61 | 50 | 0 | 220 | 80 | 0.4 | 0.2 | 0.4 | 0.4 |
| 62 | 50 | 0 | 220 | 100 | 0.4 | 0.2 | 0.4 | 0.4 |
| 63 | 50 | 0 | 220 | 120 | 0.4 | 0.2 | 0.4 | 0.4 |
| 64 | 50 | 20 | 180 | 80 | 0.4 | 0.2 | 0.4 | 0.4 |
| 65 | 50 | 20 | 180 | 100 | 0.4 | 0.2 | 0.4 | 0.4 |
| 66 | 50 | 20 | 180 | 120 | 0.4 | 0.2 | 0.4 | 0.4 |
| 67 | 50 | 20 | 200 | 80 | 0.4 | 0.2 | 0.4 | 0.4 |
| 68 | 50 | 20 | 200 | 100 | 0.4 | 0.2 | 0.4 | 0.4 |
| 69 | 50 | 20 | 200 | 120 | 0.4 | 0.2 | 0.4 | 0.4 |
| 70 | 50 | 20 | 220 | 80 | 0.4 | 0.2 | 0.4 | 0.4 |
| 71 | 50 | 20 | 220 | 100 | 0.4 | 0.2 | 0.4 | 0.4 |
| 72 | 50 | 20 | 220 | 120 | 0.4 | 0.2 | 0.4 | 0.4 |
| 73 | 50 | 40 | 180 | 80 | 0.4 | 0.2 | 0.4 | 0.4 |
| 74 | 50 | 40 | 180 | 100 | 0.4 | 0.2 | 0.4 | 0.4 |
| 75 | 50 | 40 | 180 | 120 | 0.4 | 0.2 | 0.4 | 0.4 |
| 76 | 50 | 40 | 200 | 80 | 0.4 | 0.2 | 0.4 | 0.4 |
| 77 | 50 | 40 | 200 | 100 | 0.4 | 0.2 | 0.4 | 0.4 |
| 78 | 50 | 40 | 200 | 120 | 0.4 | 0.2 | 0.4 | 0.4 |
| 79 | 50 | 40 | 220 | 80 | 0.4 | 0.2 | 0.4 | 0.4 |
| 80 | 50 | 40 | 220 | 100 | 0.4 | 0.2 | 0.4 | 0.4 |
| 81 | 50 | 40 | 220 | 120 | 0.4 | 0.2 | 0.4 | 0.4 |

Table S2: 83 combinations with different baseline parameters, achieved using full factorial design.

| Combinations | Print Speed (mm/s) | Infill Density (%) | Print Temperature (℃) | Flow Rate  (mm^3^/s) | Wall thickness (mm) | Layer height (mm) | Top Thickness (mm) | Bottom Thickness (mm) |
| --- | --- | --- | --- | --- | --- | --- | --- | --- |
| 1 | 10 | 0 | 180 | 80 | 0.4 | 0.2 | 0.4 | 0.4 |
| 2 | 10 | 0 | 180 | 100 | 0.4 | 0.2 | 0.4 | 0.4 |
| 3 | 10 | 0 | 180 | 120 | 0.4 | 0.2 | 0.4 | 0.4 |
| 4 | 10 | 0 | 200 | 80 | 0.4 | 0.2 | 0.4 | 0.4 |
| 5 | 10 | 0 | 200 | 100 | 0.4 | 0.2 | 0.4 | 0.4 |
| 6 | 10 | 0 | 200 | 120 | 0.4 | 0.2 | 0.4 | 0.4 |
| 7 | 10 | 0 | 220 | 80 | 0.4 | 0.2 | 0.4 | 0.4 |
| 8 | 10 | 0 | 220 | 100 | 0.4 | 0.2 | 0.4 | 0.4 |
| 9 | 10 | 0 | 220 | 120 | 0.4 | 0.2 | 0.4 | 0.4 |
| 10 | 10 | 20 | 180 | 20 | 0.4 | 0.2 | 0.4 | 0.4 |
| 11 | 10 | 20 | 180 | 80 | 0.4 | 0.2 | 0.4 | 0.4 |
| 12 | 10 | 20 | 180 | 100 | 0.4 | 0.2 | 0.4 | 0.4 |
| 13 | 10 | 20 | 180 | 120 | 0.4 | 0.2 | 0.4 | 0.4 |
| 14 | 10 | 20 | 200 | 80 | 0.4 | 0.2 | 0.4 | 0.4 |
| 15 | 10 | 20 | 200 | 100 | 0.4 | 0.2 | 0.4 | 0.4 |
| 16 | 10 | 20 | 200 | 120 | 0.4 | 0.2 | 0.4 | 0.4 |
| 17 | 10 | 20 | 220 | 50 | 0.4 | 0.2 | 0.4 | 0.4 |
| 18 | 10 | 20 | 220 | 80 | 0.4 | 0.2 | 0.4 | 0.4 |
| 19 | 10 | 20 | 220 | 100 | 0.4 | 0.2 | 0.4 | 0.4 |
| 20 | 10 | 20 | 220 | 120 | 0.4 | 0.2 | 0.4 | 0.4 |
| 21 | 10 | 40 | 180 | 80 | 0.4 | 0.2 | 0.4 | 0.4 |
| 22 | 10 | 40 | 180 | 100 | 0.4 | 0.2 | 0.4 | 0.4 |
| 23 | 10 | 40 | 180 | 120 | 0.4 | 0.2 | 0.4 | 0.4 |
| 24 | 10 | 40 | 200 | 80 | 0.4 | 0.2 | 0.4 | 0.4 |
| 25 | 10 | 40 | 200 | 100 | 0.4 | 0.2 | 0.4 | 0.4 |
| 26 | 10 | 40 | 200 | 120 | 0.4 | 0.2 | 0.4 | 0.4 |
| 27 | 10 | 40 | 220 | 80 | 0.4 | 0.2 | 0.4 | 0.4 |
| 28 | 10 | 40 | 220 | 100 | 0.4 | 0.2 | 0.4 | 0.4 |
| 29 | 10 | 40 | 220 | 120 | 0.4 | 0.2 | 0.4 | 0.4 |
| 30 | 30 | 0 | 180 | 80 | 0.4 | 0.2 | 0.4 | 0.4 |
| 31 | 30 | 0 | 180 | 100 | 0.4 | 0.2 | 0.4 | 0.4 |
| 32 | 30 | 0 | 180 | 120 | 0.4 | 0.2 | 0.4 | 0.4 |
| 33 | 30 | 0 | 200 | 80 | 0.4 | 0.2 | 0.4 | 0.4 |
| 34 | 30 | 0 | 200 | 100 | 0.4 | 0.2 | 0.4 | 0.4 |
| 35 | 30 | 0 | 200 | 120 | 0.4 | 0.2 | 0.4 | 0.4 |
| 36 | 30 | 0 | 220 | 80 | 0.4 | 0.2 | 0.4 | 0.4 |
| 37 | 30 | 0 | 220 | 100 | 0.4 | 0.2 | 0.4 | 0.4 |
| 38 | 30 | 0 | 220 | 120 | 0.4 | 0.2 | 0.4 | 0.4 |
| 39 | 30 | 20 | 180 | 80 | 0.4 | 0.2 | 0.4 | 0.4 |
| 40 | 30 | 20 | 180 | 100 | 0.4 | 0.2 | 0.4 | 0.4 |
| 41 | 30 | 20 | 180 | 120 | 0.4 | 0.2 | 0.4 | 0.4 |
| 42 | 30 | 20 | 200 | 80 | 0.4 | 0.2 | 0.4 | 0.4 |
| 43 | 30 | 20 | 200 | 100 | 0.4 | 0.2 | 0.4 | 0.4 |
| 44 | 30 | 20 | 200 | 120 | 0.4 | 0.2 | 0.4 | 0.4 |
| 45 | 30 | 20 | 220 | 80 | 0.4 | 0.2 | 0.4 | 0.4 |
| 46 | 30 | 20 | 220 | 100 | 0.4 | 0.2 | 0.4 | 0.4 |
| 47 | 30 | 20 | 220 | 120 | 0.4 | 0.2 | 0.4 | 0.4 |
| 48 | 30 | 40 | 180 | 80 | 0.4 | 0.2 | 0.4 | 0.4 |
| 49 | 30 | 40 | 180 | 100 | 0.4 | 0.2 | 0.4 | 0.4 |
| 50 | 30 | 40 | 180 | 120 | 0.4 | 0.2 | 0.4 | 0.4 |
| 51 | 30 | 40 | 200 | 80 | 0.4 | 0.2 | 0.4 | 0.4 |
| 52 | 30 | 40 | 200 | 100 | 0.4 | 0.2 | 0.4 | 0.4 |
| 53 | 30 | 40 | 200 | 120 | 0.4 | 0.2 | 0.4 | 0.4 |
| 54 | 30 | 40 | 220 | 80 | 0.4 | 0.2 | 0.4 | 0.4 |
| 55 | 30 | 40 | 220 | 100 | 0.4 | 0.2 | 0.4 | 0.4 |
| 56 | 30 | 40 | 220 | 120 | 0.4 | 0.2 | 0.4 | 0.4 |
| 57 | 50 | 0 | 180 | 80 | 0.4 | 0.2 | 0.4 | 0.4 |
| 58 | 50 | 0 | 180 | 100 | 0.4 | 0.2 | 0.4 | 0.4 |
| 59 | 50 | 0 | 180 | 120 | 0.4 | 0.2 | 0.4 | 0.4 |
| 60 | 50 | 0 | 200 | 80 | 0.4 | 0.2 | 0.4 | 0.4 |
| 61 | 50 | 0 | 200 | 100 | 0.4 | 0.2 | 0.4 | 0.4 |
| 62 | 50 | 0 | 200 | 120 | 0.4 | 0.2 | 0.4 | 0.4 |
| 63 | 50 | 0 | 220 | 80 | 0.4 | 0.2 | 0.4 | 0.4 |
| 64 | 50 | 0 | 220 | 100 | 0.4 | 0.2 | 0.4 | 0.4 |
| 65 | 50 | 0 | 220 | 120 | 0.4 | 0.2 | 0.4 | 0.4 |
| 66 | 50 | 20 | 180 | 80 | 0.4 | 0.2 | 0.4 | 0.4 |
| 67 | 50 | 20 | 180 | 100 | 0.4 | 0.2 | 0.4 | 0.4 |
| 68 | 50 | 20 | 180 | 120 | 0.4 | 0.2 | 0.4 | 0.4 |
| 69 | 50 | 20 | 200 | 80 | 0.4 | 0.2 | 0.4 | 0.4 |
| 70 | 50 | 20 | 200 | 100 | 0.4 | 0.2 | 0.4 | 0.4 |
| 71 | 50 | 20 | 200 | 120 | 0.4 | 0.2 | 0.4 | 0.4 |
| 72 | 50 | 20 | 220 | 80 | 0.4 | 0.2 | 0.4 | 0.4 |
| 73 | 50 | 20 | 220 | 100 | 0.4 | 0.2 | 0.4 | 0.4 |
| 74 | 50 | 20 | 220 | 120 | 0.4 | 0.2 | 0.4 | 0.4 |
| 75 | 50 | 40 | 180 | 80 | 0.4 | 0.2 | 0.4 | 0.4 |
| 76 | 50 | 40 | 180 | 100 | 0.4 | 0.2 | 0.4 | 0.4 |
| 77 | 50 | 40 | 180 | 120 | 0.4 | 0.2 | 0.4 | 0.4 |
| 78 | 50 | 40 | 200 | 80 | 0.4 | 0.2 | 0.4 | 0.4 |
| 79 | 50 | 40 | 200 | 100 | 0.4 | 0.2 | 0.4 | 0.4 |
| 80 | 50 | 40 | 200 | 120 | 0.4 | 0.2 | 0.4 | 0.4 |
| 81 | 50 | 40 | 220 | 80 | 0.4 | 0.2 | 0.4 | 0.4 |
| 82 | 50 | 40 | 220 | 100 | 0.4 | 0.2 | 0.4 | 0.4 |
| 83 | 50 | 40 | 220 | 120 | 0.4 | 0.2 | 0.4 | 0.4 |

Table S3: Physical dimensions of printlets using batch printer; 3% defects.

| **Combinations** | **Print Speed (mm/s)** | **Infill Density (%)** | **Print Temperature (℃)** | **Flow Rate (mm^3^/s)** | **Probability** | **Height (mm)** | **Diameter (mm)** |
| --- | --- | --- | --- | --- | --- | --- | --- |
| C1 | 50 | 10 | 200 | 110 | 0.8604 | 4.8 | 9.99 |
| C2 | 40 | 20 | 200 | 110 | 0.8179 | 4.81 | 10 |
| C3 | 10 | 40 | 210 | 110 | 0.7782 | 4.81 | 10 |
| C4 | 40 | 0 | 180 | 120 | 0.7312 | 4.81 | 10 |
| C5 | 30 | 20 | 190 | 110 | 0.6547 | 4.81 | 9.99 |
| C6 | 20 | 10 | 190 | 120 | 0.5628 | 4.81 | 10 |

Table S4: Physical dimensions of printlets using batch printer; 10% defects.

| Combinations | Print Speed (mm/s) | Infill Density (%) | Print Temperature (℃) | Flow Rate (mm^3^/s) | Probability | Height (mm) | Diameter (mm) |
| --- | --- | --- | --- | --- | --- | --- | --- |
| C1 | 30 | 0 | 200 | 80 | 0.8195 | 4.8 | 9.99 |
| C2 | 10 | 0 | 220 | 80 | 0.7926 | 4.8 | 9.99 |
| C3 | 40 | 0 | 200 | 80 | 0.7569 | 4.8 | 9.99 |
| C4 | 20 | 20 | 180 | 80 | 0.6837 | 4.8 | 9.99 |
| C5 | 50 | 20 | 220 | 80 | 0.6191 | 4.8 | 9.99 |
| C6 | 20 | 0 | 200 | 80 | 0.5784 | 4.8 | 9.99 |

Table S5: Physical dimensions of printlets using continuous printer: 1% defects.

| Combinations | Print Speed (mm/s) | Infill Density (%) | Print Temperature (℃) | Flow Rate (mm^3^/s) | Probability | Height (mm) | Diameter (mm) |
| --- | --- | --- | --- | --- | --- | --- | --- |
| C1 | 35 | 0 | 195 | 115 | 0.8751 | 4.78 | 9.99 |
| C2 | 40 | 25 | 205 | 80 | 0.8536 | 4.77 | 9.99 |
| C3 | 35 | 35 | 200 | 115 | 0.8078 | 4.78 | 10 |
| C4 | 25 | 25 | 215 | 110 | 0.7151 | 4.78 | 9.99 |
| C5 | 20 | 0 | 195 | 115 | 0.6891 | 4.78 | 9.99 |
| C6 | 40 | 10 | 215 | 90 | 0.5811 | 4.78 | 9.98 |

| 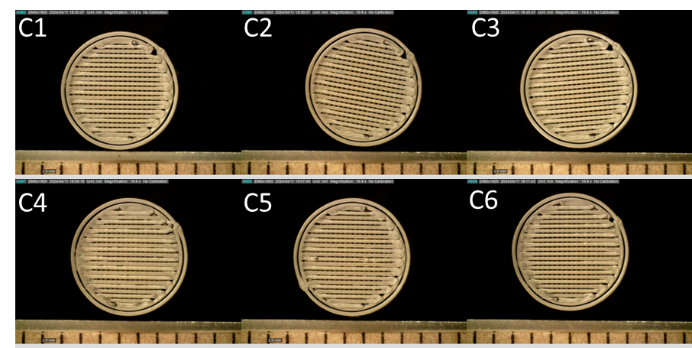  A. | 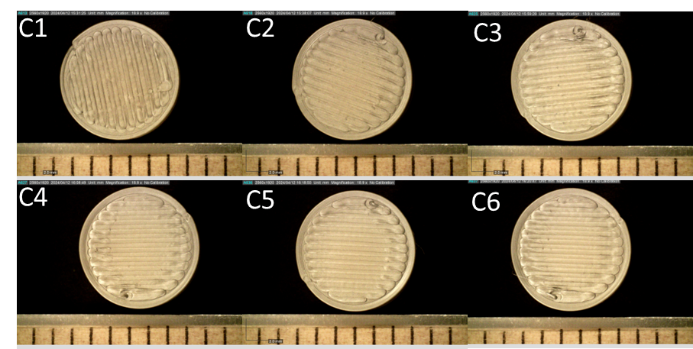  B. |
| --- | --- |

**Fig. 1S: Predicted printlets using batch printer: [A]** 10% Defects **[B]** 3% Defects


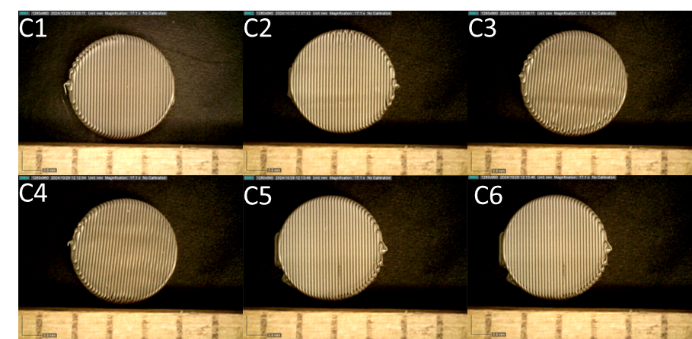


**Fig. 2S: Predicted printlets using continuous printer:** 1% Defects

Table S6: Breaking force of dogbone structures with different materials and printers

| Materials | Breaking force (N) (Batch Printer) | Breaking force (N) (Continious Printer) |
| --- | --- | --- |
| Polylactic acid (PLA) | 444.42±14.60 | 528.48±7.95 |
| Polyvinyl alcohol (PVA) | 71.99±0.46 | 64.02±1.16 |
| Thermoplastic urethane (TPU) | 53.04±0.79 | 63.99±1.04 |


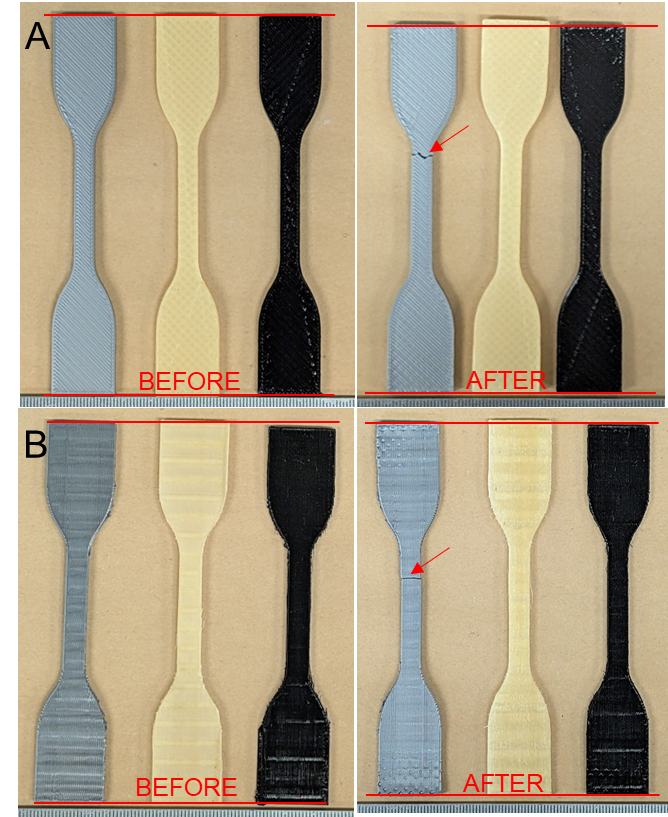


**Fig. S3: Dog bone structures before and after texture analysis** [A] Samples printed using the batch printer [B] Samples printed using the continuous printer. The red lines showing the deformation observed before and after the analysis.


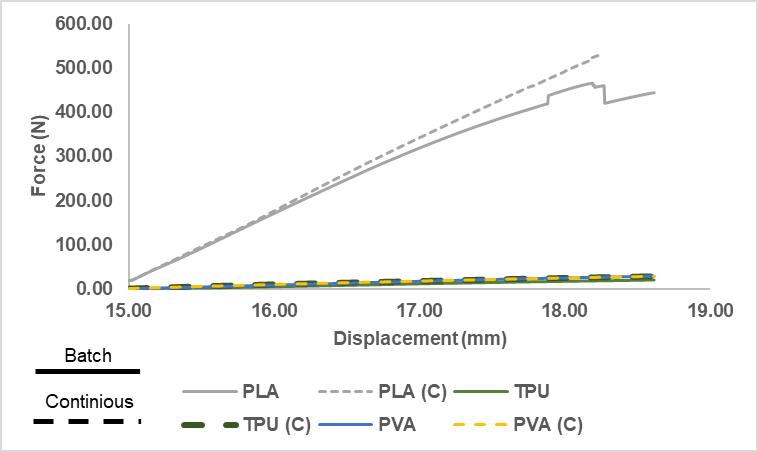


**Fig. S4**: Tensile strength analysis of dog bone structures printed with different materials
